# Modeling the load of SARS-CoV-2 virus in human expelled particles during coughing and speaking

**DOI:** 10.1371/journal.pone.0241539

**Published:** 2020-10-30

**Authors:** Yang Wang, Guang Xu, Yue-Wern Huang

**Affiliations:** 1 Department of Civil, Architectural and Environmental Engineering, Missouri University of Science and Technology, Rolla, MO, United States of America; 2 Department of Mining and Nuclear Engineering, Missouri University of Science and Technology, Rolla, MO, United States of America; 3 Department of Biological Sciences, Missouri University of Science and Technology, Rolla, MO, United States of America; Stellenbosch University, SOUTH AFRICA

## Abstract

Particle size is an essential factor when considering the fate and transport of virus-containing droplets expelled by human, because it determines the deposition pattern in the human respiratory system and the evolution of droplets by evaporation and gravitational settling. However, the evolution of virus-containing droplets and the size-dependent viral load have not been studied in detail. The lack of this information leads to uncertainties in understanding the airborne transmission of respiratory diseases, such as the COVID-19. In this study, through a set of differential equations describing the evolution of respiratory droplets and by using the SARS-CoV-2 virus as an example, we investigated the distribution of airborne virus in human expelled particles from coughing and speaking. More specifically, by calculating the vertical distances traveled by the respiratory droplets, we examined the number of viruses that can remain airborne and the size of particles carrying these airborne viruses after different elapsed times. From a single cough, a person with a high viral load in respiratory fluid (2.35 × 10^9^ copies per ml) may generate as many as 1.23 × 10^5^ copies of viruses that can remain airborne after 10 seconds, compared to 386 copies of a normal patient (7.00 × 10^6^ copies per ml). Masking, however, can effectively block around 94% of the viruses that may otherwise remain airborne after 10 seconds. Our study found that no clear size boundary exists between particles that can settle and can remain airborne. The results from this study challenge the conventional understanding of disease transmission routes through airborne and droplet mechanisms. We suggest that a complete understanding of the respiratory droplet evolution is essential and needed to identify the transmission mechanisms of respiratory diseases.

## Introduction

The ongoing pandemic of COVID-19 highlights the urgent need to understand the transport and evolution of pathogen-containing aerosols and droplets, because there are contradictory evidence and conclusions on the potential transmission route of SARS-CoV-2 [1–7]. At the very beginning of the disease outbreak, the World Health Organization (WHO) [8] and Centers for Disease Control and Prevention (CDC) [9] stated that the transmission of SARS-CoV-2 through the airborne route, which is by inhaling virus-containing aerosols, is unlikely. Instead, droplet transmission, which is through exposure to respiratory droplets, and contact transmission, which is the infection through direct or indirect contact with an infected person, are believed to be the major transmission routes. The traditional distinction between a “droplet” and an “aerosol (or droplet nuclei)” is based on size, where droplets are suspended particles above 5 μm in diameter, and aerosols are those below 5 μm [10]. To avoid confusion, in this study, we will use “particles” to refer to a summation of “aerosols” and “droplets.” It is thought that droplets can settle to ground in a few seconds, but aerosols can remain airborne for an extended period of time. Although there is no such definition in atmospheric studies, this traditional distinction between droplets and aerosols has been useful for setting clinical guidelines on the use of personal protective equipment for healthcare workers [11]. However, the conventional distinction between aerosols and droplets has led to a “false dichotomy” [12] in understanding airborne pathogens, because whether a respiratory particle can remain airborne depends on many factors.

Existing studies show that human activities such as coughing, sneezing, and speaking generate particles, with more than 90% of the total particle numbers less than 5 μm after evaporation [13–17]. Evaporation can significantly extend the dispersion lifetime of particles before they settle, enhancing the infection risk of airborne viruses. For example, the sizes of the largest droplets that would totally evaporate before settling 2 m are between 60 and 100 μm, and these expelled large droplets are carried more than 6 m away by exhaled air at a velocity of 50 m s^-1^ (sneezing), more than 2 m away at a velocity of 10 m s^-1^ (coughing) and less than 1 m away at a velocity of 1 m s^-1^ (breathing) [14]. Many of these existing studies, including a recent one [18] investigated the droplet lifetime influenced by the ambient temperature and humidity using the evaporating drop mathematical model, but the virus contained in the particles, and the associated viral load as a function of particle size were not included in the model. This particle size-dependent viral load is crucial to our understanding of the relative importance of airborne and droplet transmission because if a significant number of viruses remain in airborne, appropriate precautions should be taken, such as universal masking, stronger indoor ventilation rate, and air disinfection. Until now, more evidence is also showing that similar to other pathogens such as influenza viruses and Mycobacterium tuberculosis [19], SARS-CoV-2 can be carried by aerosols [20–25].

Theoretically, coughing, sneezing, and speaking generate particles by aerosolizing the respiratory fluid, and the number of viruses in a particle is determined by the viral concentration in the respiratory fluid and the volume of the particle. Therefore, the number of viruses in a single particle should scale with the cube of the particle diameter. Based on the typical concentration of the SARS-CoV-2 viruses in respiratory fluid [26], one can calculate that a considerable number of human expelled particles do not contain viruses due to their small volume. During the evolution of the respiratory droplets, evaporation complicates the size-dependent viral load in aerosols and droplets, as the size of the particles changes with time. Gravitational settling will remove larger droplets that contain more viruses. Collectively, they ensure the necessity to examine the load of viruses in human expelled particles of different sizes.

Using the most recent SARS-CoV-2 data, this study used the Monte-Carlo method to simulate the particles generated from coughing and speaking and used a Poisson distribution function to determine the virus load in the particles. The particle size-dependent viral load and its variation as a function of time during evaporation and gravitational settling are modeled using mass and heat transfer equations and the momentum equation. The detailed modeling methods are elaborated in the Methods section. In the Results and Discussion section, we show that most of the virus-containing particles can remain airborne for an extended period of time longer than 10 seconds. We analyzed how the elapsed time and viral load in the respiratory fluid affect the transport of the virus-containing particles, and examined the particle emission from coughing and speaking. Finally, we discusses the uncertainties associated with this analysis.

## Methods

### Size distributions of human expelled particles

Accurate size distributions of human expelled droplets are required to estimate the particle size-dependent viral load. Existing studies commonly used an Aerodynamic Particle Sizer (APS, TSI Inc.) to measure the size distributions of human-emitted droplets [16, 27–29]. However, droplets will evaporate during their transport in the measurement setup, leading to uncertainties in measuring the original droplet sizes. The size distributions of directly emitted droplets can be more accurately measured by in-situ light scattering experiments conducted near the human mouth [30, 31]. In this study, we adopted such droplet size distributions measured by Chao et al. [30], where speaking generates particles with a geometric mean diameter (*D*_d,g_) of 16.0 μm and a geometric standard deviation (*σ*_d,g_) of 0.55, and coughing generates particles with a *D*_d,g_ of 13.5 μm and a *σ*_d,g_ of 0.50. We further assume that speaking and coughing generate a total number (*N*_d_) of 50 per second and 3000 per cough, respectively [16, 30]. The droplet size (*D*_d_) follows a lognormal size distribution, where

$$n_{d}\left(D_{d}\right)=\frac{N_{d}}{\sqrt{2\pi }\ln\;\left(\sigma_{d,g}\right)}\exp\;\left[-\frac{\left(\ln\left(D_{d}\right)-\ln\left(D_{d,g}\right)\right)}{2{\left(\ln\left(\sigma_{d,g}\right)\right)}^{2}}\right].$$ (1)

We adopted a Monte-Carlo method to randomly generate *N*_d_ number of droplets following the lognormal size distribution. The number of viruses in a droplet with a size of *D*_d_ can be calculated by

$$\mathrm{VL}\left(D_{d}\right)=\frac{\pi }{6}D_{d}^{3}C_{V},$$ (2)

where *C*_V_ is the viral load of SARS-CoV-2 in the respiratory fluid. Existing studies show that *C*_V_ has an average value of 7.00 × 10^6^ copies per ml, with a maximum of 2.35 × 10^9^ copies per ml, which is largely dependent on the number of days after onset of symptoms [32]. We further assume that the liquid content of the respiratory fluid is composed of 0.9% NaCl-water solution. Therefore, after evaporation, the expelled particles can leave a solid core containing salt and viruses, which is a more realistic model of respiratory particles.

We should note that the number of viruses calculated by Eq (2) is hardly an integer. VL(*D*_d_) reflects the expected number of viruses in a droplet, but the actual number will take integer values above or below VL(*D*_d_). To reflect the randomness of this process, we assume that the actual number of viruses enclosed in a droplet follows the Poisson distribution [33]. We have

$$f\left(x\right)=\frac{{\left(\mathrm{VL}\left(D_{d}\right)\right)}^{x}}{x!}\exp\;\left[-\mathrm{VL}\left(D_{d}\right)\right].$$ (3)

In this equation, *f*(*x*) is the probability the droplet with a size *D*_d_ containing exactly *x* (*x* = 0,1,2,…) number of viruses.

### Evaporation and gravitational settling

After being emitted, a droplet undergoes evaporation and gravitational settling. The size of the droplet is determined by the following mass and heat transfer equations:

$${\dot{m}}_{d}=\rho_{d}\frac{d}{dt}\left(\frac{\pi }{6}D_{d}^{3}\right)=-A_{d}h_{m}\left(p_{v,s}-p_{v,\infty }\right),\;\mathrm{and}$$ (4)

$$m_{d}C_{\mathrm{Pd}}\frac{dT_{d}}{dt}=A_{d}h\left(T_{\infty }-T_{d}\right)+L{\dot{m}}_{d}.$$ (5)

The droplet evaporation rate ${\dot{m}}_{d}$ is driven by the difference between the vapor pressure in the surrounding air *p*_v,∞_ and the vapor pressure at the droplet surface *p*_v,s_. *p*_v,s_ is assumed as saturated vapor pressure at droplet temperature *T*_d_, considering the Kelvin and Raoult effects. *A*_d_ is the droplet surface area, *L* is the latent heat of vaporization, and *C*_Pd_ is the heat capacity of the droplet. The mass transfer coefficient *h*_m_ and the heat transfer coefficient *h* can be solved using the Ranz-Marshall correlations for the Sherwood and Nusselt numbers [34]. The ultimate droplet size is determined by the solid components in the droplet. Previous studies on respiratory droplet evaporation commonly ignored the influence of microorganisms enclosed in the droplet, leading to an underestimate of the final particle size and overestimate of the particle lifetime. In this model simulation, we further considered the influence of SARS-CoV-2 on the physical size of the evaporated droplet, by assuming that the enclosed SARS-CoV-2 virus has a spherical shape and diameter of 100 nm (65 to 125 nm according to Astuti et al. [35]) and a density of 1.35 g cm^-3^, similar to common protein [36].

The gravitational settling of the human expelled particles can be solved by the momentum balance equation, where

$$m_{d}\frac{d^{2}z}{dt^{2}}=\frac{1}{2}\rho_{g}V_{z}^{2}A_{d}C_{D}.$$ (6)

In Eq (6), *z* is the droplet settling distance, *ρ*_g_ is ambient air density, *V*_z_ is droplet velocity in the vertical direction, *A*_d_ is the cross section area of the droplet ($A_{d}=\frac{\pi }{4}D_{d}^{2}$), and *C*_D_ is the drag coefficient, which is dependent on the Reynolds number of the particle motion [37]. In this study, we focus on the vertical movement of the particles in order to estimate whether the particles can remain airborne after different elapsed time. The horizontal movement of the particles will largely depend on the activity that generates the particles, and they will be examined briefly at the end of the analyses.

The differential equations in Eqs (4–6) can be solved simultaneously, where the droplet diameter, droplet surface temperature, and droplet settling distance can be derived as a function of time. Assuming that these human expelled droplets are generated at the height of 1.7 m with no initial vertical velocity, we can further calculate the lifetime of a droplet, which is the time corresponding to *z* = 1.7 m. For all the calculations, we assume an indoor environmental condition, where the temperature is 23 ^o^C and the relative humidity is 50%. Conceivably, temperature and relative humidity can affect the droplet evolution through evaporation, as shown in Chen 2020 [18]. Moreover, they will likely influence the viability of viruses and, thereby the infection risk [38], which is discussed at the end of the following section. However, this study focuses on modeling the number of viruses that can remain airborne after being emitted by the patient. We should note that there are other modeling methods available to understand the dynamics associated with biological and physical systems [39–41]. In this work, we used the relatively simplified differential equations to understand the transport of the virus-containing aerosols and estimate the load of viruses in human expelled particles.

## Results and discussion

In the following analysis, we demonstrate how the airborne viral load depends on the size of the human expelled particles and its variation as a function of time. We first analyze the load of the airborne virus on particles generated from a single cough, and then examine its dependence on elapsed time and the viral load in the respiratory fluid. We also compare the airborne viral load associated with speaking against that of coughing.

### Droplet properties at the point of emission

**Fig 1:**
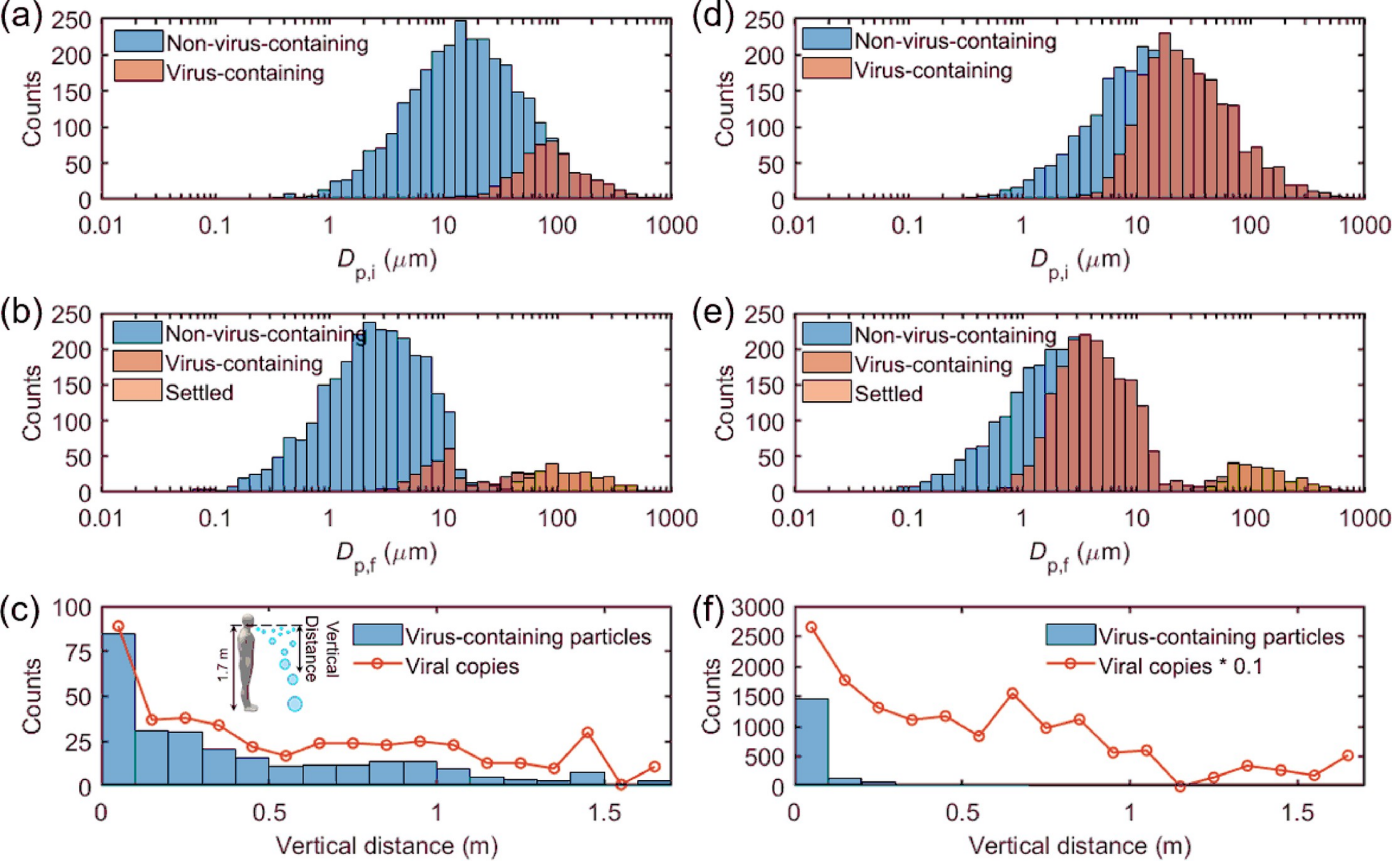
Evolution of droplets emitted by a cough over an elapsed time of ten seconds at respiratory viral loads of (a–c) 7.00 × 10^6^ and (d–f) 2.35 × 10^9^ copies per ml. (a) and (d) Size distribution of droplets and virus-containing droplets at point of emission. (b) and (e) Size distribution of non-virus-containing (airborne), virus-containing (airborne), and settled particles at an elapsed time of ten seconds. (c) and (f) Distribution of vertical distances traveled by the virus-containing particles at an elapsed time of ten seconds. The inset figure in panel (c) shows a schematic of the modeled system.

Fig 1 shows an example solution demonstrating the evolution of droplets generated during a single cough. Fig 1A displays the size distribution of 3000 coughing droplets randomly generated following the lognormal distribution in Eq (1). At a viral load of 7.00 × 10^6^ copies per ml in the respiratory fluid, viruses are mostly contained in droplets larger than 10 μm, because the product of the droplet volume and the viral concentration in smaller droplets is far below 1. Among the 3000 droplets generated by a single cough, approximately 390 ± 16 droplets contain viruses, and the total number of viruses in these virus-containing droplets is 9.8 × 10^3^ ± 6.4 × 10^3^ copies (Table 1). This large standard deviation is a result of a few giant droplets, which contain a substantial number of viruses. However, these giant droplets are also subject to rapid removal by gravitational settling as time progresses.

**Table 1:** Number of virus-containing particles and number of viral copies remain suspended in the air after different elapsed times in a cough.

	Viral load in respiratory fluid (copies per ml)	
	7.00 × 10^6^	2.35 × 10^9^
Virus-containing droplets after 0 s	390 ± 16	2021.6 ± 22.4
Viral copies after 0 s	9.8 × 10^3^ ± 6.4 × 10^3^	2.6 × 10^6^ ± 1.7 × 10^6^
Virus-containing particles after 1 s	380 ± 6	2017 ± 25
Viral copies after 1 s	4.4 × 10^3^ ± 0.7 × 10^3^	1.33 × 10^6^ ± 0.11 × 10^6^
Virus-containing particles after 3 s	349 ± 16	1990 ± 23
Viral copies after 3 s	1.2 × 10^3^ ± 0.1 × 10^3^	4.15 × 10^5^ ± 0.11 × 10^5^
Virus-containing particles after 10 s	250 ± 7	1855 ± 13
Viral copies after 10 s	386 ± 7	1.23 × 10^5^ ± 0.05 × 10^5^
Virus-containing particles after 30 s	232 ± 14	1871 ± 7
Viral copies after 30 s	333 ± 12	1.13 × 10^5^ ± 0.01 × 10^5^

### Effect of elapsed time

After ten seconds of evaporation and gravitational settling, the peak size of the expelled particles shifted to around 2.2 μm (Fig 1B). Due to the salt and viruses in the droplet, the virus-containing particles now have a size above 2 μm. Approximately 5.1% of virus-containing particles are below 5 μm, which traditionally would be categorized as "aerosols.” The number of viruses contained in these sub-5 μm particles is 20 ± 2 copies. However, 59.5% of virus-containing particles remain airborne (settle less than 1.7 m), and the number of viruses contained in the evaporated droplets is 386 ± 7 copies. This result shows that one cannot simply use a specific size to determine whether a respiratory particle settle or remain airborne. Droplet evaporation and heat transfer over time need to be incorporated to be more accurately depict the respiratory particle behavior. Fig 1C also shows the vertical distance traveled by the virus-containing particles and the number of viruses contained in the particles after ten seconds of droplet emission. It demonstrates that around 80% of the virus-containing particles settle with a vertical distance within 0.5 m, meaning that these suspended particles can linger in the inhalation zone of people surrounding the patient.

The number of viral copies contained in the particles decreases rapidly with the elapsed time, from 9.8 × 10^3^ at the point of emission to 333 ± 12 at an elapsed time of 30 s. It is because larger particles that enclose more viral copies settle faster (Fig 1B). On the other hand, the number of virus-containing particles that remain airborne is relatively insensitive to elapsed time, from 390 ± 16 at the point of emission to 232 ± 14 at 30 s. This insensitivity is caused by the fact that most of the virus-containing droplets shrink to sizes that cannot be effectively settled by gravity. Therefore, these particles will have a longer lifetime and pose a higher infection risk.

### Effect of viral load in respiratory fluid

**Fig 2:**
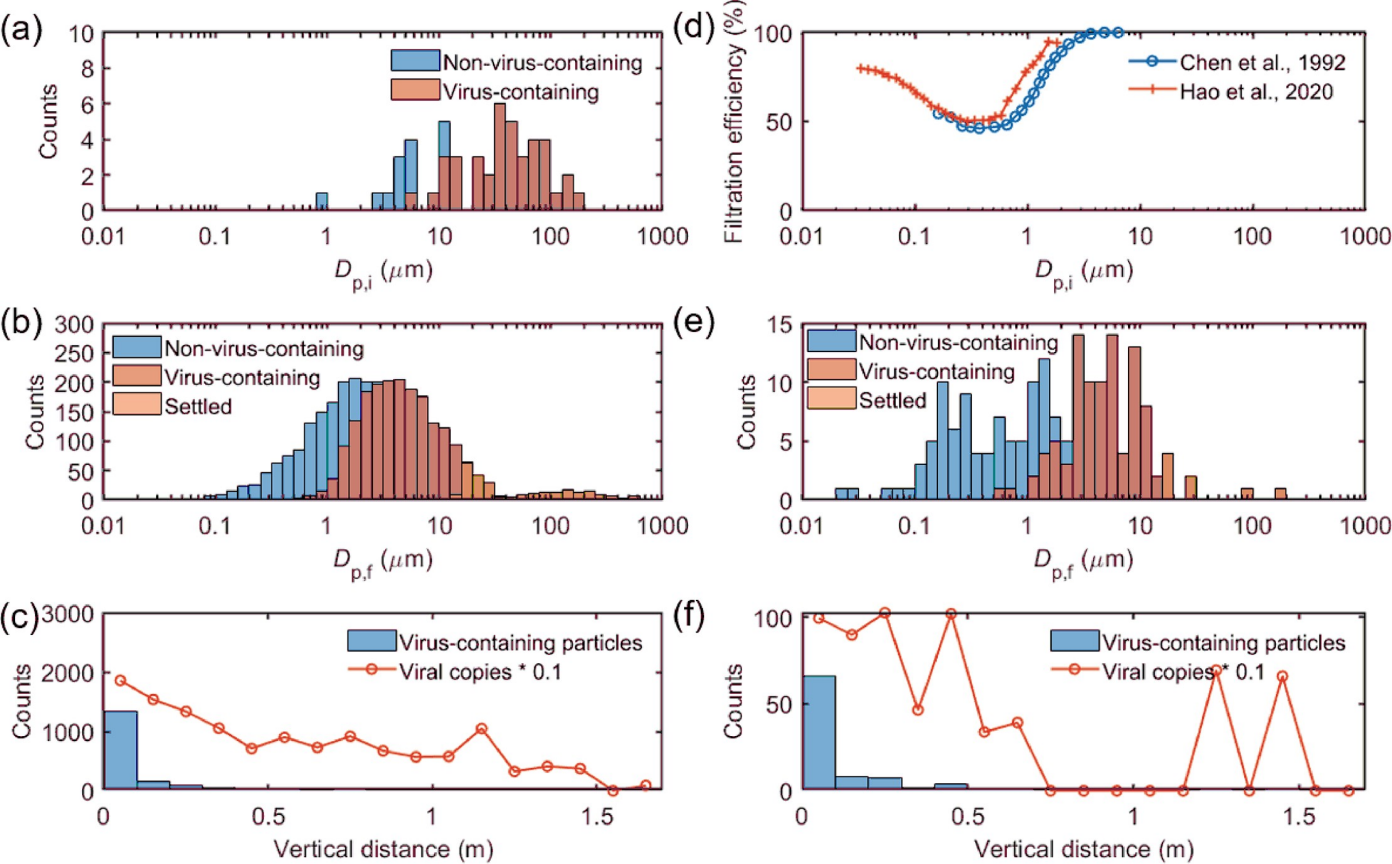
Evolution of droplets emitted by one-minute of speaking after an elapsed time of ten seconds at a respiratory viral load of 2.35 × 10^9^ copies per ml. (a) Size distribution of droplets and virus-containing droplets at point of emission during one-second of speaking. (b) Size distribution of non-virus-containing (airborne), virus-containing (airborne), and settled particles at an elapsed time of ten seconds. (c) Distribution of vertical distances traveled by the virus-containing particles at an elapsed time of ten seconds. (d) Size-dependent filtration efficiency curves for a surgical mask (earloop) extracted from Chen et al. [42] and Hao et al. [43]. (e) Size distribution of non-virus-containing (airborne), virus-containing (airborne), and settled particles at an elapsed time of ten seconds with mask-wearing. (f) Distribution of vertical distances traveled by the virus-containing particles at an elapsed time of ten seconds with mask-wearing.

The viral load in the respiratory fluid drastically affects the evolution of human expelled virus-containing particles (Fig 1D–1F). At a viral load of 2.35 × 10^9^ copies per ml, droplets as small as 4 μm start to contain viruses (Fig 1D), and around 67.4% of droplets contain viruses. The fraction of virus-containing particles remaining airborne after an elapsed time of ten seconds is also high (Fig 1E), reaching 61.8%. Again, it is not realistic to use a cut-off size of 5 μm to differentiate “aerosols” from “droplets.” Due to the high viral load in the respiratory fluid (2.35 × 10^9^ copies per ml), the number of viral copies in the evaporated particles (1.23 × 10^5^) is orders of magnitude higher compared to the average condition (386 under 7.00 × 10^6^ copies per ml). The vertical distribution of the virus-containing particles and the copies of viruses in Fig 2F show considerably higher values in shorter vertical distances (0 to 0.5 m), meaning that a patient with a higher viral load in the respiratory fluid would pose a significantly higher infection risk to the surrounding people.

### Airborne viral load during speaking

Compared to coughing, speaking is a process that continuously generated respiratory droplets. Therefore, when examining the evolution of droplets as a function of time, we need to consider the droplets emitted at different times of speaking cumulatively. Fig 2A shows the properties of droplets during one second of speaking at the point of emission for a patient with a viral load of 2.35 × 10^9^ copies per ml in the respiratory fluid. Due to the few numbers of droplets generated, the droplet size distribution is subject to high uncertainty. Fig 2B shows the size distribution of speaking-generated particles ten seconds after a one-minute speech. The size distribution is not significantly different from that of coughing, as shown in Fig 1E. However, due to the longer elapsed time of particles emitted at the beginning of the speaking period (up to 70 seconds), particles of 20 μm can settle down to the ground, compared to 40 μm for coughing. However, the vertical distribution of the numbers of virus-containing droplets and viral copies still show higher numbers in shorter vertical distances (0 to 0.5 m), meaning that a considerable fraction of speaking-generated droplets can remain airborne due to evaporation.

### Effect of mask-wearing

Using the proposed model, we could also evaluate the effectiveness of face masks in preventing the spread of viruses. Fig 2D shows the size-dependent filtration efficiency of aerosols from 0.03 to 10 μm for common surgical mask materials [42, 43]. Due to the combined mechanisms of inertial impaction, interception, Brownian diffusion, and electrostatic interaction, the filtration efficiency curves generally show an “escape window” where particles with hundreds of nanometers can penetrate through the filter, resulting in lower efficiencies. Existing literature also uses the term “most penetrating particle size (MPPS)” to describe the reduced filtration efficiency in this size range [44]. Unlike medical respirators, face masks have the issue of flow leakage between the mask and the wearer [45]. Here, we assume a flow leakage of 5%, and calculated the evolution of droplets generated from speaking using the average filtration efficiency in Fig 2D. The numbers of both the non-virus-containing and virus-containing droplets reduced significantly (Fig 2E) compared to the unmasked speaking (Fig 2B), with the total number of airborne virus-containing droplets decreased by 94.9% (from 2122 ± 17 to 108 ± 5), and with the total number of viral copies decreased by 95.6% (from 1.4 × 10^5^ ± 0.1 × 10^5^ to 6.2 × 10^3^ ± 0.2 × 10^3^). Although the number of virus-containing particles is still the highest near the point of emission (within the vertical distance of 0.5 m, Fig 2F), the number of viral copies decreased significantly within this distance. Due to the effective removal of virus-containing particles, the vertical distribution of the number of viral copies becomes more random, and the two peaks in the distance between 1 and 1.7 m in Fig 2F are caused by a few large droplets that escaped from the mask. Compared to the unmasked condition (Fig 2B), the number fraction of evaporated particles below 1 μm becomes higher under the masked condition (Fig 2E), mainly due to the lower filtration efficiencies of the masks for particles between 0.1 and 1 μm.

### Uncertainties associated with the analysis

The above analysis shows that a significant fraction of respiratory droplets can remain airborne after they are emitted. Note that the horizontal movement of the droplets is not shown in this study, because the horizontal velocity of respiratory droplets depends strongly on human activity, age, and ambient environment [46–48]. The trajectory of the exhaled respiratory droplets is affected by both the expired air flows profile and surrounding air flow patterns. Existing studies treated the exhaled air as a turbulent round jet [49, 50], and the turbulent flow will enhance the heat and mass transfer between the droplet and the surrounding air. Therefore, respiratory droplets will likely evaporate faster than the simulated results in this study, and a larger fraction of respiratory droplets and viruses may remain airborne for a longer period of time. Here, we adopt a simplified flow field derived from a previous experimental study [51], where the horizontal velocity of air expelled from coughing follows the equation

$$V_{x}=0.875/{\left(l_{x}+0.333\right)}^{2}.$$ (7)

In Eq (7), *V*_x_ is the velocity of the respiratory droplet in the horizontal direction in m s^-1^ when there is no ambient air flow and *l*_x_ is the horizontal distance from the point of emission in m. According to this relationship, the distance traveled by the respiratory droplets as a function of time can be derived as:

$$l_{x}=\sqrt[{3}]{\left(2.625t+0.0369\right)}-0.333.$$ (8)

According to this simplified solution, airborne droplets can travel a horizontal distance of 2.64 m after 10 s, and 3.95 m after 30 s. Considering that virus-containing particles can remain airborne after 30 seconds (Table 1), the “six-feet (or 2 m) rule” is not sufficient in preventing disease transmission. Nonetheless, universal masking may be a better option for disease transmission, as it can capture the respiratory droplets effectively through impaction and interception at the source of generation [43, 52].

In this study, we did not consider the viability of viruses in particles with different sizes. Since pathogen viability is dependent on the surface properties of materials [53], the viability of viruses in droplets may also change as a function of time, because evaporation continuously increases the droplet surface tension and expose the components of the droplet to the surface of the droplet. For example, virus deactivation may occur after exposure to the air-water interface, where irreversible rearrangement and folding of the viruses’ protein take place [54, 55]. Moreover, the distribution of viruses in droplets of different sizes may not be uniform. For example, studies on airborne virus sampling show that viable viruses tend to be sampled in particles below 5 μm [56, 57]. One possible explanation is that droplets of different sizes may originate from different regions of the respiratory system, where smaller droplets are formed from regions of a higher viral load. The measurement of virus-laden aerosols in outbreaks in farms also indicated that certain viruses tend to be associated with particles below 0.4 μm [58], which may be due to the mechanism of aerosol generation. Therefore, future work can futher study how the expired air flows and size-dependent viability of the viruses affect the concentration of the airborne viruses generated from coughing and speaking.

## Conclusion

In this work, we investigated the dependence of airborne viral load on the size distributions of the human expelled particles. We found that differentiating “aerosols” and “droplets” using a specific size, e.g., 5 μm, does not reflect the actual evolution of virus-containing particles over time and space, because a large number of particles above 5 μm can remain airborne after an extended period of time. Our simulation result showed that after ten seconds of a cough, although most evaporated particles are larger than 5 μm, 59.5% of the original virus-containing particles are still able to remain airborne. Although the numbers of airborne viral copies and virus-containing particles decrease with elapsed time, this dependence becomes weaker at long elapsed times due to the significantly longer residence time of the smaller particles. We further show that a high viral load in the respiratory fluid will lead to a significantly higher infection risk due to the large number of virus-containing aerosols that remain airborne after an extended elapsed time. Our simulation also shows that wearing a mask can effectively reduce the spread of the viruses. The simulation results challenge the false dichotomy of using aerosols and droplets to separate the modes of disease transmission.
